# Transcriptome profiling of the feeding-to-fasting transition in chicken liver

**DOI:** 10.1186/1471-2164-9-611

**Published:** 2008-12-17

**Authors:** Colette Désert, Michel J Duclos, Pierre Blavy, Frédéric Lecerf, François Moreews, Christophe Klopp, Marc Aubry, Frédéric Herault, Pascale Le Roy, Cécile Berri, Madeleine Douaire, Christian Diot, Sandrine Lagarrigue

**Affiliations:** 1 INRA, UMR 598, Génétique Animale, F-35000 Rennes, France; 2 Agrocampus Ouest, UMR 598, Génétique Animale, F-35000 Rennes, France; 3 INRA, UR83, Station de Recherches Avicoles, F-37380 Nouzilly, France; 4 INRA, SIGENAE, F-35000 Rennes, France; 5 INRA, SIGENAE, F-31000 Toulouse, France; 6 Plateforme Transcriptome OUEST-genopole Rennes, F-35000 Rennes, France

## Abstract

**Background:**

Starvation triggers a complex array of adaptative metabolic responses including energy-metabolic responses, a process which must imply tissue specific alterations in gene expression and in which the liver plays a central role. The present study aimed to describe the evolution of global gene expression profiles in liver of 4-week-old male chickens during a 48 h fasting period using a chicken 20 K oligoarray.

**Results:**

A large number of genes were modulated by fasting (3532 genes with a pvalue corrected by Benjamini-Hochberg < 0.01); 2062 showed an amplitude of variation higher than +/- 40% among those, 1162 presented an human ortholog, allowing to collect functional information. Notably more genes were down-regulated than up-regulated, whatever the duration of fasting (16 h or 48 h). The number of genes differentially expressed after 48 h of fasting was 3.5-fold higher than after 16 h of fasting. Four clusters of co-expressed genes were identified by a hierarchical cluster analysis. Gene Ontology, KEGG and Ingenuity databases were then used to identify the metabolic processes associated to each cluster. After 16 h of fasting, genes involved in ketogenesis, gluconeogenesis and mitochondrial or peroxisomal fatty acid beta-oxidation, were up-regulated (cluster-1) whereas genes involved in fatty acid and cholesterol synthesis were down-regulated (cluster-2). For all genes tested, the microarray data was confirmed by quantitative RT-PCR. Most genes were altered by fasting as already reported in mammals. A notable exception was the *HMG-CoA synthase 1 *gene, which was up-regulated following 16 and 48 h of fasting while the other genes involved in cholesterol metabolism were down-regulated as reported in mammalian studies. We further focused on genes not represented on the microarray and candidates for the regulation of the target genes belonging to cluster-1 and -2 and involved in lipid metabolism. Data are provided concerning PPARa, SREBP1, SREBP2, NR1H3 transcription factors and two desaturases (FADS1, FADS2).

**Conclusion:**

This study evidences numerous genes altered by starvation in chickens and suggests a global repression of cellular activity in response to this stressor. The central role of lipid and acetyl-CoA metabolisms and its regulation at transcriptional level are confirmed in chicken liver in response to short-term fasting. Interesting expression modulations were observed for *NR1H3, FADS1 *and *FADS2 *genes. Further studies are needed to precise their role in the complex regulatory network controlling lipid metabolism.

## Background

All animal species have evolved a metabolic response system allowing them to survive during periods of energy deprivation. The overall metabolic response to fasting operates at numerous levels and has been relatively well characterized in mammals [1-6]. In vertebrates, the liver plays a central role in this adaptive response. Deprivation of food inhibits lipogenesis and induces the release of large amounts of fatty acids from the adipose tissue, which are taken up by the liver and oxidized in the peroxisome and/or mitochondria via beta-oxidation. The majority of fatty acids are only partially oxidized to acetyl-coenzyme A (acetyl-CoA), which then condenses with itself to form ketone bodies, an important fuel for the brain. The energy released in the process of beta-oxidation is used by the liver to carry out gluconeogenesis from substrates such as glycerol, lactate, and amino acids.

Several studies showed that the inhibition of fatty acid synthesis and the induction of gluconeogenesis, ketogenesis and fatty acid beta-oxidation in response to fasting result from changes in mRNA level of genes encoding enzymes and transcription regulators involved in these metabolisms [6]. Several studies using PPARa-null mice [7-9] have demonstrated a key role of PPARa in this response. Although microarray-based experiments have been widely used to identify differentially expressed genes involved in numerous biological processes, only few studies have considered the effect of fasting on large-scale hepatic gene profiles. Data are available in the mouse [10,11], the pig [12] (but the study was limited to 1272 cDNA) and more recently the rainbow trout [13] and the rat [14]. In the present study, the chicken species was chosen as an important model organism that bridges the evolutionary gap between mammals and other vertebrates; a divergence that occurred about 300 million years ago. Previous studies have shown that the activities or expression of hepatic enzymes involved in lipogenesis, beta-oxidation and gluconeogenesis (ME, ACLY, ACACA [15]; CPT1A, EHHADH [16]; PEPCK [17]) and plasma metabolites and hormones levels (Glucose, lactate, pyruvate, aceto-acetate, B-hydroxybutyrate [17]; Insulin [18]) were altered during fasting in chickens. Overall the available data show that chickens share most metabolic responses with mammals despite some distinct features. In birds lipogenesis occurs essentially in the liver [19-21] contrary to rodents or pigs in which it is regulated in both liver and adipose tissue. Regulation of gluconeogenesis differs too, essentially due to intracellular location of key enzymes [22]. Chicken plasma metabolites have different levels from those reported for mammals, especially glucose which is higher. To extend and complete previous studies conducted on candidate genes, the present study aimed to describe the evolution of global gene expression profiles in chicken liver during a 48 h fasting period by using pangenomic oligo microarrays. The second reason to choose chicken as model is that this species provides a major protein source from meat throughout the world and the excessive accumulation of lipids in birds is one of the main problems facing this industry. To our knowledge, no whole genome survey of hepatic gene expression has been reported in chickens so far [23]. The use of a recently available 20 K oligo microarray allowed us to make the first description of the alteration of hepatic gene profiles induced by fasting in this species and make some hypothesis on the regulatory mechanisms involved at the mRNA level. The genes showing a significant alteration in their expression profile during fasting were grouped in four clusters of co-expressed genes by a two-way Hierarchical Clustering Analysis. Further interpretation was based on the use of different gene annotation databases (GO, KEGG and Ingenuity databases) highlighting numerous biological processes modulated by fasting.

## Results

### Experimental and microarray setup

To evaluate the changes in hepatic gene expression in response to starvation, 4 week-old male chickens were submitted to 0, 16 h or 48 h of food withdrawal. Transcriptome profiling of these "Fed", "Fst16" and "Fst48" nutritional stages was carried out by using a 20 K microarray. The expression data of the 20460 gene set from 18 independent oligochips was normalized by "Lowess-fitness". Of the 20460 oligos present on the microarray, 13057 aligning with a unique coding region of the 2.1 Washington University assembly of the chicken sequence genome, were chosen for statistical analyses. The expression data were further analyzed by analysis of variance with the eBayes procedure [24] using LIMMA package (see Methods) to identify differentially expressed genes between either of the two fasting states and the fed state. Clusters of co-expressed genes were identified by two-way Hierarchical Cluster Analysis and the degree of importance of each gene in this cluster identification was explored by Principal Component Analysis. Gene Ontology, KEGG and INGENUITY databases were used for further interpretation, as 7419 genes (out of 13057) presented a human ortholog with a HUGO symbol allowing us to recover functional annotations from those databases.

The microarray data results were deposited in the Gene Expression Omnibus (GEO) public repository . The accession number for the series is GSE11290 and the sample series can be retrieved with accession number GSM278021, GSM282110, and GSM284914 up to GSM284948. The sample series contains for each microarray the raw data (median signal) of each Cy5 and Cy3 channels as well as the normalized data (log2(ratio Cy5/Cy3))

### A high number of genes differentially expressed between the two fasting states and the Fed state

**Figure 1:**
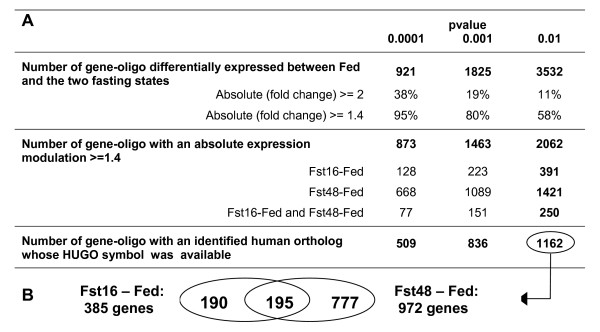
**Genes differentially expressed between Fed and the two fasting Fst16 and Fst48 states**. A: Number of genes differentially expressed according to the pvalues corrected by Benjamini-Hochberg. B: Venn diagram for the genes differentially expressed (pvalue < 0.01) in the two contrasts "16 h of fasting versus the fed state" (Fst16-Fed) and "48 h of fasting versus the fed state" (Fst48-Fed).

A high number of gene transcripts were significantly altered by fasting, so that we considered only those presenting an amplitude of variation higher than +/- 40% between fasting and fed conditions (Figure 1A). The percentage of genes presenting such a modulation increased lower pvalues were choosen, for example for a cut-off fixed to 1.4, it increased from 58% with a pvalue of 0.01 to 95% with a pvalue of 0.0001. In the present study, the 2062 genes that satisfied the criterion of pvalue < 0.01 and absolute fold-change > 1.4 were considered for further analysis. Among these genes, the number of regulated genes was 3.5-fold higher after 48 h of fasting than after 16 h. We further performed a global analysis of the 1162 genes fulfilling those conditions and for which annotation could be retrieved through their human ortholog (Figure 1-B). The selected 0.01 pvalue that was corrected according to the false discovery rate (FDR) of Benjamini-Hochberg [25] ensures in average a number of false positive of 12 genes in this selected gene set. In this set, 190 genes were altered after 16 h of fasting, 777 after 48 h of fasting, and 195 after 16 h and 48 h (with the concordant variations for 190 of them).

**Figure 2:**
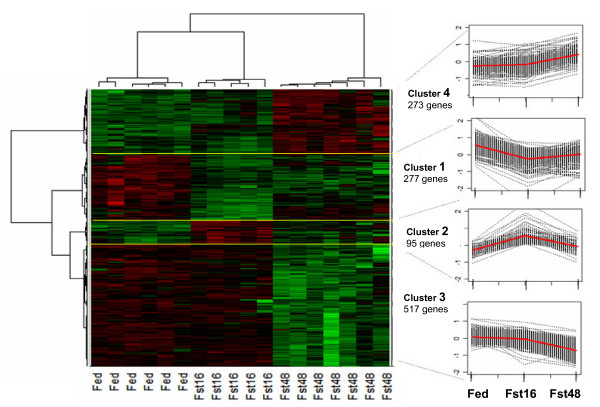
**Two-way Hierarchical Cluster Analysis (HCA) of the gene selection**. On the right, for each cluster, the curves of the expression of all the genes corresponding to the three nutritional states (the mean curve is in red).

A two-way Hierarchical Cluster Analysis (HCA) was applied to the normalized dataset to identify clusters among the 18 animals and these 1162 genes (Figure 2). As expected, the animal dendrogram clearly separated the three nutritional conditions: the Fst48 state was more distant from the Fed state than the Fst16 state. The dendrogram also showed a higher homogeneity between individuals intra than inter nutritional conditions, showing that the number of chickens analyzed by condition was appropriate for such a study.

For the gene variables, four clusters were identified. The clusters 1 (277 genes) and 2 (95 genes) were respectively down- and up-regulated after 16 h of fasting. The majority of genes in cluster 1 (66%) were also significantly down-regulated after 48 h of fasting, but with an amplitude similar to that observed after 16 h. The majority of genes in cluster 2 (71%) returned to fed levels by 48 h of fasting. The clusters 3 (517 genes) and 4 (273 genes) corresponded to the genes respectively down- and up-regulated only after 48 h of fasting. Altogether, the number of genes significantly down-regulated by fasting was higher than the number of genes up-regulated: 799 versus 360 genes. Only 3 genes (*TMEM43, AP3B2, C1orf59*) were regulated in an opposite way between the Fst16 and Fst48 states. The list of the 1162 significantly differentially expressed genes with the associated cluster is presented in an additional file 1.

**Figure 3:**
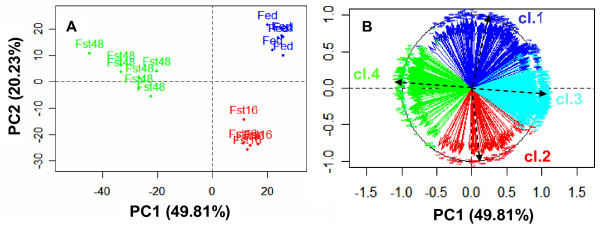
**Principal Component Analysis (PCA) of the gene selection**. The gene variables for the PCA were scaled to give them the same importance. A: individual factor map; B: gene factor map, only the genes highly correlated to the first two components are indicated (r > 0.7). The gene color was determined according to their HCA cluster (cl.i = cluster i).

In complement to the HCA, Principal Components Analysis (PCA) allowed to identify the genes contributing most to the separation of individuals. The first two components of PCA (explaining 70% of the total variability) clearly separated the three nutritional conditions (Figure 3-A) and about 80% of the genes showed a high correlation with the two principal components (> 0.7) (Figure 3-B). Further analysis of the whole dataset was then considered to identify the main biological processes regulated by fasting.

### Annotation of gene clusters using Gene Ontology (GO), KEGG and Ingenuity databases

Gene ontology (GO) terms have been widely used as a tool for a global interpretation of microarray data. Table 1 indicates the biological process GO terms enriched for each of the 4 clusters obtained by HCA (pvalue < 0.01). The most over-represented GO terms (high pvalue and at least 3 genes associated to the term) concerned "lipid biosynthesis", "fatty acid biosynthesis" corresponding to genes down-regulated after 16 h of fasting (cluster 1) and "fatty acid beta-oxidation" and "gluconeogenesis" corresponding to genes up-regulated after 16 h of fasting (cluster 2). These observations were consistent with those already reported in mammals.

**Table 1:** Annotation of gene clusters using Gene Ontology (GO), KEGG and Ingenuity databases

	**Biological process GO terms **^1^	**KEGG pathways **^2^	**Ingenuity pathways **^3^
**Cluster 1**	**Lipid metabolism** SLC27A4 -LYPLA2-ENPP6-DGAT2L4-CYP51A1-FASN-SULT4A1-CPNE7-ANGPTL3-ACACA-LSS-ACLY-PBX1-PIGH-PLCL1-PRKAG3-PRKAA2-SCD -SBF2-PLA2G12B-MTMR3-PITPNM1 P = 5.E-04	**Fatty acid biosynthesis** ACLY-FASN-ACACA-SCD	**Fatty Acid Biosynthesis** FASN-ACACA-MCCC2 P = 5.E-04
**277 genes**	**Lipid biosynthesis** DGAT2L4-CYP51A1-FASN-ACACA-LSS-ACLY-PBX1-PIGH-PRKAG3-PRKAA2-SCD P = 2.E-03 **Fatty acid biosynthesis** FASN-ACACA-PRKAG3-PRKAA2-SCD P = 6.E-03 **Regulation of action potential** KCNMB2-SBF2-EIF2B4 P = 7.E-03		

**Cluster 2** **95 genes**	**Lipid metabolism** PECI-DCTN6-CPT1A-FABP1-ACSL1-ACAA1-HADHA-HMGCS1-APOB-ACOX1-SCP2-ADIPOR2-PLCZ1 P = 3.E-06 **Fatty acid metabolism** PECI-CPT1A-FABP1-ACSL1-ACAA1-HADHA-ACOX1-ADIPOR2 P = 2.E-07 **Fatty acid oxidation** CPT1A-HADHA-ACOX1-ADIPOR2 P = 4.E-06 **Fatty acid beta-oxidation** CPT1A-HADHA-ACOX1 P = 2.E-05 **Energy derivation by oxidation of organic compounds** FBP1-IDH1-PCK1-FBP2-GYG2 P = 8.E-03 **Gluconeogenesis** FBP1-PCK1-FBP2 P = 4.E-04	**Fatty acid metabolism** ACAT1-PECI-ACAA1-ACOX1-HADHA-ACSL1 **PPAR signalling pathway** PCK1-SCP2-FABP1-ACAA1-ACOX1-ACSL1 **Synthesis and degradation of ketone bodies** ACAT1-HMGCL-ACAA1-HMGCS1 **Citrate cycle TCA cycle** PCK1-ACAT1-HMGCL-IDH1-ACAA1 **Gluconeogenesis** PCK1-ACAT1-HMGCL-FBP1 **Valine.leucine and isoleucine degradation** ACAT1-HMGCL-ACAA1-HADHA-HMGCS1 **Lysine degradation** ACAT1-BBOX1-HADHA	**Fatty Acid Metabolism** CYP3A43-CYP4A22-CPT1A-ACAA1-CYP2C18-ACOX1-PECI-ACAT1-ACSL1-HADHA P = 2.E-11 **Synthesis and Degradation of Ketone Bodies** 1-06E01 6-42E-02 CYP3A43-CYP4A22-CPT1A-ACAA1-CYP2C18-ACOX1-PECI-CPT2-ACAT1-EHHADH-ACSL1-HADHA ACAA1-ACAT1-HMGCL-HMGCS1-HADHA P = 2.E-11 **Pyruvate Metabolism** ACAA1-ACAT1-PCK1-ACSL1-HADHA P = 2.E-05 **Valine- Leucine and Isoleucine Degradation** ACAA1-ACAT1-HMGCL-HMGCS1-HADHA P = 9.E-08 6-94E00 6-54E-02 ACAA1-ACAT1-HMGCL-HMGCS2-EHHADH-HMGCS1-HADHA **Lysine Degradation** BBOX1-ACAA1-ACAT1-MLL3-HADHA P = 1.E-06 **Tryptophan Metabolism** CYP3A43-CYP4A22-ACAA1-CYP2C18-ACAT1-HADHA P = 8.E-06 **Propanoate Metabolism** ACAA1-ACAT1-ACSL1-HADHA P = 1.E-05 1-36E00 2-02E-02 EHHADH-HADHA 8-54E-01 1-69E-02 PCK1-IGFBP1

**Cluster 3** **517 genes**	**Cell cycl**e MPHOSPH9-TRIM13-TADA3L-YWHAQ-ESCO2-DBC1-E2F6-ERN1-FGF6-SH3BP4-FRAP1-GAS2-APBB2-KRAS-MAD2L1-MDM2-MLH1-MUTYH-NRAS-NDE1-PPP1CB-CDC37L1-PBK-PRKG2-PTMS-BARD1-CLSPN-NEK4-YWHAH-MAD1L1-MCM8-CCNH-PRC1-MTSS1-CUL7-DCLRE1A-CDC34 P = 9.E-03 **Glycolysis** DLAT-ENO1-OGDH-PGAM1-PKM2-UEVLD P = 8.E-03 **Cytokinesis** CECR2-PRC1-ROCK2 P = 7.E-03 **Ceramide metabolism** GALC-PRKAA1-SGPL1 P = 7.E-03 **Regulation of protein catabolism** ATE1-MDM2-BARD1 P = 2.E-03	**Insulin signalling pathway** PTPN1-PRKAA1-KRAS-FRAP1-PRKAB1-SORBS1-CRK-NRAS **ErbB signalling pathway**_ CAMK2A-JUN-KRAS-FRAP1-PAK7-SRC-CRK-NRAS- **GnRH signalling pathway** CAMK2A-ADCY3-JUN-KRAS-CACNA1C-SRC-NRAS **Renal cell carcinoma**_ JUN-SLC2A1-KRAS-PAK7-ARNT-CRK-NRAS-EGLN3 **Tight junction**_ JAM3-KRAS-CLDN16-SRC-MAGI1-NRAS-ACTB **Glioma**_ CAMK2A-KRAS-FRAP1-MDM2-NRAS	**Chemokine Signaling** ROCK2-SRC-PLCB4-CAMK2A-NRAS-JUN-MYL2-PPP1CB-KRAS P = 3.E-04 **Ephrin Receptor Signaling** MAP3K14-SRC-NRAS-CREB3-SOS2-KRAS-CRK-ROCK2-AKT1-SORBS1-EFNB1-ACP1-ARPC4-PRKAA1-PAK7-MAK P = 6.E-04 **B Cell Receptor Signaling** MAP3K14-FRAP1-AKT1-CAMK2A-NRAS-JUN-MAP3K7-CREB3-SOS2-PIK3AP1-KRAS P = 3.E-03 **Estrogen Receptor Signaling** SRC-PRKDC-CCNH-NRAS-SOS2-KRAS-GTF2A1-MED4-ESR2 P = 3.E-03 **PDGF Signaling** SRC-NRAS-JUN-ACP1-SOS2-KRAS-CRK P = 4.E-03 **Wnt/Î^2^-catenin Signaling** SRC-AKT1-WIF1-MAP3K7-WNT7B-FZD3-DKK2-MDM2 (includes EG:4193)-WNT5B-WNT2-SOX5 (includes EG:6660) P = 6.E-03 **Actin Cytoskeleton Signaling** ABI2-TIAM1-NRAS-MYL2-ACTB-SOS2-PPP1CB-KRAS-CRK-FGF6-ROCK2-ARPC4-PRKAA1-PAK7-MAK P = 6.E-03 **JAK/Stat Signaling** FRAP1-AKT1-NRAS-SOS2-PTPN1-KRAS P = 8.E-03 **Hypoxia Signaling in the Cardiovascular System** AKT1-JUN-COPS5-CREB3-MDM2 (includes EG:4193)-CDC34-UBE2I-ARNT P = 6.E-04

**Cluster 4** **273 genes**		**TGF.beta signalling pathway** ACVR2A-PPP2R2A-RBL1 **Complement and coagulation cascades** F13A1-C8B-F8-PLAU **Purine metabolism** ADSL-ITPA-ATIC-POLR1B-POLR3G-POLR2C **Gap junction** CDC2-PDGFA-MAP2K2-GNAI1 **RNA_polymerase** POLR1B-POLR3G-POLR2C **Histidine_metabolism** ADSL-GAD1-ATIC **Long.term depression** RARB-CASP9-STK4-MAP2K2 **Long.term_potentiation** GRIN2B-GRIN2A-MAP2K2	**cAMP-mediated Signaling** CNGA4-MAP2K2-GNAO1-GNAI1-HTR1A-RGS12-CNGA1-CHRM3-CHRM5 P = 1.E-03 1–69°00 6–67°-02 HDAC4-TGFB3-RBL1-SIN3A **Ephrin Receptor Signaling** GRIN2B-GRIN2A-MAP2K2-PDGFA-CXCL12-GNAO1-ITGA2-GNAI1-ARPC3-CDC2-ACVR2A P = 1.E-03 1–26°00 3–64°-02 SOX9-GNAO1-RARB-TGFB3-SFRP5-ACVR2A

The pathways sub-lined were found in at least two of the three analyses.^1 ^Biological process GO terms obtained by the Gene Ontology Tree Machine software (GOTM). Are only indicated the enriched biological process GO terms with a significant level of pvalue < 0.01 (see Methods) and a minimum of 3 genes associated. ^2 ^Kegg pathways: are only indicated those with a minimum of 3 genes associated and having a probability to be observed in the cluster 4-fold superior than the probability to obtain it by chance.^3 ^Ingenuity pathways: are indicated the top five Canonical pathways associated to each cluster (pvalue < 0.01). Only canonical pathways with at least 3 genes affiliated were conserved.

Among the enriched GO terms of the cluster 3 (down-regulated after 48 h of fasting only) were "glycolysis", "regulation of protein catabolism" and surprisingly "cell cycle" with more than 30 genes associated. Of the six genes associated with "Glycolysis", *PKM2 *encodes a pyruvate kinase that catalyses an irreversible reaction in glycolysis. *ATE1 *and *MDM2*, two genes annotated as involved in the "regulation of protein catabolism" are part of the ubiquitin-proteasome system. No enriched GO terms was found for the cluster 4.

The biological interpretation of the gene clusters was further completed using KEGG database [26] and Ingenuity Pathway Analysis (IPA, Ingenuity Systems Inc., Redwood City, CA). The significant KEGG and IPA pathways are indicated in Table 1 with the genes associated. IPA and Kegg pathway analyses highlighted some biological processes already revealed by GO analysis, plus other ones, showing the interest in combining different sources of annotation. Cluster 1, down-regulated at 16 h of fasting, was mainly characterized by genes involved in cholesterol metabolism and fatty acid biosynthesis, through the annotation terms "lipid metabolism", "lipid biosynthesis", "carboxylic acid biosynthesis", "fatty acid biosynthesis". Lipogenic genes (*ACACA, FASN, SCD, ACLY*), cholesterogenic genes (*CYP51A1, LSS*) and genes involved in triglyceride synthesis (*DGAT2L4, ANGPTL2*) were found in the list. Cluster 2, up-regulated at 16 h of fasting, was essentially characterized by genes involved in fatty acid metabolism/oxidation and acetyl-CoA metabolism through the annotation terms "fatty acid beta-oxydation", "energy derivation by oxidation of organic compounds", "gluconeogenesis", "PPAR signalling pathway", "synthesis and degradation of ketone bodies", "citrate cycle TCA cycle" and "pyruvate metabolism". The list comprised genes involved in fatty acid beta-oxidation (*PECI, ACAA1, ACOX1, CPT1A, HADHA*), ketogenesis (*HMGCL, ACAT1*), gluconeogenesis (*PCK1, FBP1, FBP2*) and fatty acid transport or activation (*ACSL1, APOB, FABP1*). Cluster 3, down-regulated at 48 h of fasting, was essentially characterized by genes involved in "glycolysis" (6 genes), "cell cycle" (more than 30 genes associated to this GO term) and 12 signalling pathways including "Insulin signaling pathway" and "Estrogen Receptor Signaling". Cluster 4, up-regulated at 48 h of fasting, was associated to various annotation terms, notably "cAMP-mediated Signaling" linked to glucagon/insulin balance.

Following this global overview of the data, we chose to focus on genes regulated by the first 16 hours of fasting (clusters 1 and 2) that were involved in "lipid biosynthesis", "fatty acid beta-oxidation", "ketogenesis" and "gluconeogenesis". Several genes were chosen for validation of the microarray results using quantitative RT-PCR. A further group of genes encoding FADS1 and FADS2 desaturases and transcriptional factors known to be important regulators of hepatic lipid metabolism were also considered.

### Genes involved in fatty acid- and cholesterol-synthesis and in fatty acid beta-oxidation, ketogenesis and gluconeogenesis

#### Validation by qRT-PCR

**Figure 4:**
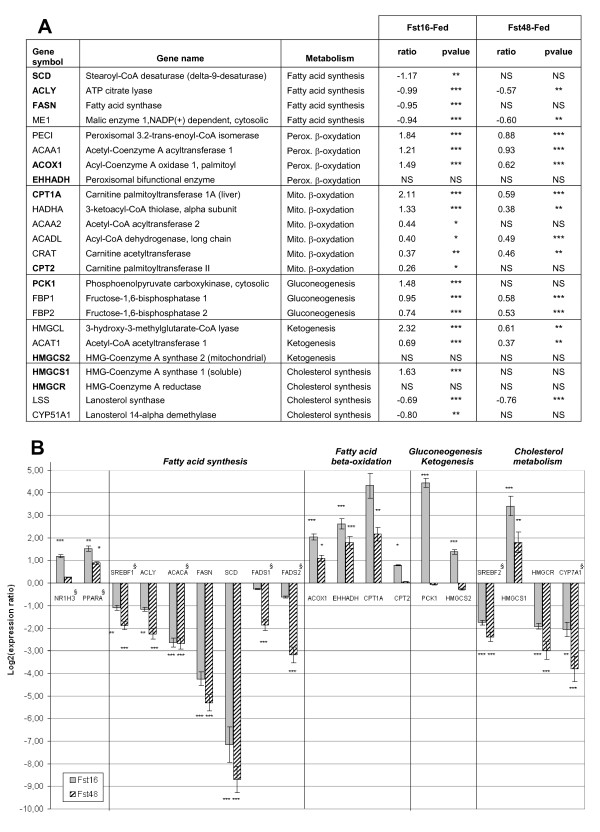
**Identification of differentially expressed genes involved in fatty acid synthesis, beta-oxidation, ketogenesis, gluconeogenesis and cholesterol metabolism**. A: *Expression of genes present on microarray and analyzed by microarray procedure*. Gene symbol: HGNC Hugo abbreviation of the human ortholog of the Gallus gallus gene represented by the oligo spotted on microarray. Results were expressed as a log2 ratio of the gene expression between fasted and fed states ("Fst16-Fed" or "Fst48-Fed" contrasts). Genes in gold were analyzed by qRT-PCR (see B). P values were corrected by Benjamini-Hochberg (see Methods). * pvalue < 0.05, ** pvalue < 0.01, *** pvalue < 0.001, NS: non-significant. B: *Validation of microarray results by qRT-PCR*. PCRs were realized in triplicate with SyBr Green and specific primers for each gene (See Table 1). 18S ribosomal RNA was used as reference. Results were expressed as a log2 ratio of the gene expression for the two "Fst16-Fed" and "Fst48-Fed" contrasts. Statistical significance is indicated as following: *pvalue < 0.05, **pvalue < 0.01, ***pvalue < 0.001. Genes not analyzed by microarray procedure are indicated with §

Twenty four genes involved in peroxisomal or mitochondrial beta-oxidation of fatty acids, in ketogenesis, in gluconeogenesis and in fatty acid and cholesterol synthesis were present on the microarrays, respectively 10, 3, 3, 4 and 4 genes. As indicated in Figure 4-A, after 16 h of fasting, all lipogenic genes and cholesterogenic genes were significantly down-regulated, except *HMGCR *no regulated and *HMGCS1 *up-regulated. By contrast, all genes involved in ketogenesis, gluconeogenesis and mitochondrial or peroxisomal fatty acid beta-oxidation were significantly up-regulated, except *EHHADH *and *HMGCS2*. The highest amplitudes of up-regulations were observed for *CPT1A, PECI, ACAA1, ACOX1, HMGCL, PCK1 *(~2- up to 4-fold, pval < 10^-8^). The genes *ACLY, FASN, ME1 *and *SCD *were down-regulated by 2-fold. To confirm these microarray data, 8 of the 21 genes differentially expressed after 16 h of fasting were analyzed by qRT-PCR (Figure 4-B). The results confirmed the microarray analyses, but higher amplitudes of variation were generally measured by qRT-PCR. Notably, the up-regulation of the *HMGCS1 *gene during fasting was largely confirmed (3-fold by microarray and 10-fold by quantitative RT-PCR). The 3 genes *HMGCR, EHHADH *and *HMGCS2 *not found significantly differentially expressed by microarray were analyzed by quantitative RT-PCR as well. *EHHADH *was up-regulated during fasting (16 h or 48 h) and HMGCR down-regulated, as described in mammals. By contrast, *HMGCS2 *was found significantly up-regulated but only after 16 h of fasting. Overall these results are consistent with those reported in mammals except for the gene *HMGCS1*, which could show species specific patterns of regulation.

#### Expression of genes encoding SREBP-1, PPARa, SREBP-2 and NR1H3 transcription factors

Because Peroxysome Proliferators-Activated Receptor alpha (PPARa), Sterol Regulatory Element Binding Protein 1 and 2 (SREBP1 and SREBP-2) and Nuclear Receptor Subfamily 1, group H, member 3 (NR1H3 also noted LXRa for Liver X receptor alpha), have been identified in mammals as critical transcription factors for the regulation of hepatic fatty acid beta-oxidation, fatty acid synthesis or cholesterol metabolism [6], we measured their expression by quantitative RT-PCR (Figure 4-B). *PPARa*: We observed an up-regulation of *PPARa *in chicken livers after fasting (3- and 2-fold at Fst16 and Fst48 states respectively). The correlations at the mRNA level between *PPARa *and its putative targets in chickens (*ACOX1, EHHADH, CPT1, HMGCS2*) were significantly high: 0.82 to 0.86 using the qRT-PCR expressions of the 18 chickens in Fed, Fst16 and Fst48 states. Using the 2.1 Washington University assembly of the Gallus gallus genome sequence, the upstream sequences of those genes were screened for a putative PPARa response element (PPRE). As indicated in Table 2, potential PPRE were identified in *ACOX1, CPT1 *and *HMGCS2*. *SREBP1*: The first 16 h of fasting caused a significant repression of the hepatic expression of *SREBP1 *(2- and 4-fold for Fst16 and Fst48 states respectively). The correlations between *SREBP1 *and its putative targets in chickens (*ACLY, ACACA, FASN *and *SCD*) at the mRNA level were significantly high (0.87 to 0.92 using the 18 chickens in Fed, Fst16 and Fst48 states). *SREBP2*: The transcriptional factor SREBP2 regulates the expression of many genes involved in cholesterol synthesis. We observed by qRT-PCR a decrease of *SREBP2 *gene expression after fasting, (4- and 6-fold after 16 h and 48 h of fasting) correlated to *HMGCR *gene expression. Microarray results also indicated a 2-fold decreased expression of genes encoding CYP51A1 (Lanosterol 14alpha-demethylase) and LSS (Lanosterol synthase), other enzymes involved in cholesterol synthesis. By contrast, *HMGCS1 *was strongly up-regulated during starvation. *NR1H3*: *NR1H3 *mRNA level was significantly increased (by 2-fold) after 16 h of fasting and remained unchanged after 48 h whereas genes known to be its direct targets in mammals were down-regulated by fasting. It was the case for the lipogenic genes *SREBP1, FASN *and *SCD *and for *Cyp7a1 *(cholesterol 7 hydroxylase) encoding the rate-limiting enzyme of cholesterol degradation to bile acids.

**Table 2:** PPRE prediction in Gallus gallus HMGCS2, CPT1A and ACOX1 genomic DNA sequences

**Genes**	**Species**	**Ensembl ID**	**PPRE Pattern**	**Location**	**Strand**	**Methods**
**HMGCS2**	**RNO**	**Rodriguez *et al*. 1994 **[79]	**GGGCCAaAGGTCT**	Promoter region		
	GGA	ENSGALG00000002960	GGGCCAaAGGTCC	-530	+	STAN/PATSER
	HSA	ENSG00000134240	GGGTCAaAGGGCT	-118	+	STAN
	MUS	ENSMUSG00000027875	GGGCCAaAGGGAT	-104	RC	STAN

**CPT1A**	**HSA/MUS/RNO**	**Napal *et al*., 2005 **[80]	**AGGGGAaAGGTCA**	Intronic region between exon 1 and exon 2		
	GGA	ENSGALG00000007077	AGGGAAaAGGGCA	4964	+	STAN
	HSA	ENSG00000110090	AGGGGAaAGGTCA	2426	+	STAN
	MUS	ENSMUSG00000024900	AGGGGAaAGGTTA	2180	RC	STAN

**ACOX1**	**MUS**	**Tugwood *et al*., 1992 **[81]	**AGGACAaAGGTCA**	Promoter region		
	GGA	ENSGALG00000002159	-	-	-	
	HSA	ENSG00000161533	-	-	-	
	MUS	ENSMUSG00000020777	-	-	-	
	
	**RNO**	**Krey *et al*., 1995 **[82]	**AGGTAGaAGGTCA**	Promoter region		
	GGA	ENSGALG00000002159	AGGAAGaAAGTCA	-3931	+	STAN/PATSER
	HSA	ENSG00000161533	-	-	-	STAN
	MUS	ENSMUSG00000020777	AGGTAAaAGGTCA	52	+	STAN

PPRE patterns used for the research is indicated in bold with the reference associated. GGA: Gallus gallus; HSA: Homo sapiens; MUS: Mus musculus. Nucleic acids in red indicate conserved nucleic acids with the PPREs used for the research. Location: genomic location refers to the start of the Ensembl first exon (+1). Strand: "RC" indicates that the sequence is reverse complemented compared to the genomic sequence displayed at that location, otherwise a "+" is indicated. STAN or PASTER indicates that the PPRE has been identified either by STAN or PATSER softwares respectively (see Methods). For the *ACOX1 *gene, despite previous results indicating PPRE in the Mus musculus promoting DNA sequence [81], we cannot found any PPRE with the STAN procedure in any species. We used another PPRE pattern [82] identified in the Rattus norvegicus DNA sequence. This pattern allowed us to identify potential PPRE in Gallus gallus and Mus musculus sequences. The absence of result in the Homo sapiens sequence was consistent with the literature [78].

#### Expression of genes involved in desaturation of fatty acids

GO term, IPA and Kegg pathway analyses highlighted SCD which is considered as an enzyme involved in mono-unsaturated fatty acid synthesis. However, SCD (also named delta9-desaturase) belongs to the fatty acid desaturase family, which also includes FADS1 (delta5-desaturase) and FADS2 (delta6-desaturase), two key enzymes for the production of polyunsaturated fatty acids. We observed that fasting resulted in a 14- to 18-fold inhibition of *SCD *expression (measured by qRT-PCR at 16 h and 48 h of fasting respectively, Figure 4-B). A significant repression was observed for both *FADS1 *and *FADS2 *genes but only after 48 h of fasting (4- or 6-fold depending on the genes, Figure 4-B). The data show that the expression profiles of *SCD *gene and the two *FADS1 *and *FADS2 *genes were different in response to fasting in chickens.

## Discussion

To date few studies have reported global gene expression surveys in chickens. Wang et al [27] provided an analysis of the chicken adipose tissue gene expression profile using a 28 k GeneChip Chicken Genome Array (Affymetrix Inc.). Other hepatic transcriptional analyses have been reported but using only dedicated chicken microarrays: 3.2 K liver-specific microarray [28,29] or a 323 cDNA microarray [30]. Although microarray studies are more numerous in mammalian species, only few studies have considered global gene expression of the liver tissue in response to fasting. The effect of fasting has been studied in the liver of mice fasted during 24 h or 48 h using a 20 K oligo microarray [10,11] and in the liver of rats fasted during 24 h with a 30 K oligo-chip [14]. One study was conducted in the liver of pigs fasted during 24 h which used a 1272 cDNA microarray [12], and more recently one was conducted in the liver of trouts submitted to 3 weeks of food deprivation which used a 16 K microarray [13]. In the present study, we analyzed the hepatic expression of 13057 genes in 4 week-old chickens, submitted to 16 h or 48 h of starvation. Of the 7419 annoted genes on the microarray (e.g. having a human ortholog), we observed 1162 genes with an amplitude of variation higher than +/- 40% at pvalue below 0.01 (corrected for multiple test). Because our gene selection satisfied the criterion of FDR < 1% (so only 12 genes were statistically false positives) our results could be considered as reliable; and indeed all the differential genes tested were confirmed by qRT-PCR. It remains, however, that the amplitude of the differences measured by qRT-PCR were generally higher than those measured using microarrays. This may explain why *EHHADH, HMGCR *and *HMGCS2 *were observed as differentially expressed by qRT-PCR but not by microarray; and it may suggest that the microarray analyses based on hybridization technique is more hampered by proportion of false negative compared to qRT-PCR analyses because of the lack of an amplification step. The list of the 1162 genes differentially expressed in the chicken liver after starvation is available in additional file 1.

The main observation was that a high number of genes were regulated by fasting in chicken liver and in a time dependent manner: the expression of 190 genes was altered by 16 h of fasting versus that of 777 genes was altered by 48 h. In mouse liver, Bauer et al (2004) [10] made a similar observation (131 genes and 269 genes modulated by 24 h and 48 h of fasting respectively) even if the number of differential genes was lower compared to the present study. Another important observation was that the number of genes down-regulated by fasting was higher than the number of genes up-regulated, whatever the duration of fasting (16 h or 48 h): 799 genes versus 360 genes respectively. Among the 190 genes annoted as enzymes that represented 16% of the genes annoted, 136 genes (72%) were down-regulated. Numerous reports have shown that birds are relatively resistant to long period of starvation [31-34]. This is the case for chickens which resist to longer period of starvation than mice [33,34]. Four week old chickens survive starvation periods of more than 10 days [35], loosing about 37% of body weight during this period. This suggests that the severity of a 48 h fasting period is limited. However, our observations suggest a global repression of the cellular activity in response to short-term starvation (16–48 h).

Principal components analysis clearly separated the three nutritional conditions; the most (80%) of the differentially expressed genes were correlated with the two principal components and so contributed to distinguish the three states. By a two-way hierarchical clustering analysis, 4 clusters were identified: 277 genes were down-regulated and 95 genes up-regulated after 16 h of fasting, most of them showing a similar pattern at 48 h of fasting. A prolonged starvation up to 48 h repressed 517 and induced 273 gene expressions. We then used the three databases, Gene Ontology, KEGG and INGENUITY to analyze the 4 gene clusters. These databases supply a useful tool for a global biological interpretation of microarray data. We can however notice some contradictions which could be due to the imperfection of these annotations. First, a biological process could be highlighted through genes which are not key regulators of this process but only indirectly involved in it. As an illustration, the "Valine, leucine and isoleucine degradation" process was cited from KEGG and IPA databases in cluster 2 through genes involved in ketogenesis (Table 1) even though the first key enzyme of this pathway, BCAT1 (an aminotransferase) was absent of this cluster and belonged to cluster 1. Second, the allocation of a gene to a pathway may not always be optimal. For example, Gamma-butyrobetaine dioxygenase (BBOX1, cluster 2), was associated to the "Lysine degradation pathway" in KEGG and IPA databases while this enzyme catalyses carnitine biosynthesis and should therefore be associated to "fatty acid beta-oxidation" process in which carnitine is essential for mitochondrial beta-oxidation [36]. Another limitation may arise from the fact that some particular genes may not be annoted by automatic annotation processes. For instance, the Pyruvate dehydrogenase kinase 4 gene (*PDK4 *up-regulated after 16 h of fasting,) was not cited in the "gluconeogenesis" pathway associated to the cluster 2, whereas PDK4 phosphorylates and inhibits the activity of the pyruvate dehydrogenase complex and thus promotes the conversion of pyruvate into lactate, which is used in the liver for gluconeogenesis. Other genes important in the "lipid biosynthesis" process and associated to cluster 1 were also not highlighted: Insulin receptor Signaling gene (*IRS2*) or Insulin induced gene (*INSIG1*) which mediate feedback control of cholesterol and fatty acid synthesis by controlling SREBP cleavage-activating protein (SCAP) involved in the regulation of cleavage of Sterol response element binding proteins (SREBP1 and SREBP2). Considering these imperfections, the annotation results require an expert biological knowledge to validate them and draw up precise comments and this is a limit to the full interpretation of pangenomic microarray data. However, the use of such databases, in particular when several are combined as in the present study, remains a first and useful step for a global biological interpretation as it allows highlighting the major pathways involved.

In the present study, the GO, KEGG and IPA annotation results taken together are complementary and allow us to draw up the following overview about the general biological mechanisms associated with each cluster. First, and as could be expected, they point out to an alteration of lipid and acetyl-CoA metabolisms during the first 16 h of fasting (clusters 1 and 2): lipogenic and cholesterogenic genes were down-regulated whereas genes involved in fatty acid beta-oxidation, ketogenesis, gluconeogenesis and fatty acid activation or transport were up-regulated. These results are in agreement with the previously reported alterations of glucose and lipid metabolisms in response to short-term starvation in rodents [10,14,37,38], pigs [12] and chickens [15,19]. Second and less expected, they highlight a number of cell signalling pathways which would seem to be altered between 16 h and 48 h of fasting (clusters 3 and 4). Notably, genes in the "Insulin signalling cascade" would seem to be repressed whereas genes in the "cAMP-mediated signalling" pathway would seem to be enhanced. More than ten signalling response pathways were identified in cluster 3 (Table 1), suggesting a general repression of signalling pathways at 48 h of starvation. Many genes associated with these pathways are implicated in cellular responses, also involved in cell proliferation and differentiation. Precise comments about them would need an expert biological knowledge.

Because PPARa, SREBP1, SREBP-2 and NR1H3 have been identified in mammals as critical transcription factors for the regulation of fatty acid beta-oxidation, fatty acid synthesis or cholesterol metabolism, we examined their expressions by qRT-PCR.

Definite proof that PPARa plays a key role in the up-regulation of fatty acid beta-oxidation, ketogenesis and gluconeogenesis in the liver during starvation comes from several studies using PPARa-null (KO) mice. Using a gene-by-gene approach, these studies identified PPARa target genes [7-9,39]. Short-term fasting (12–72 h) in KO mice resulted in hepatic steatosis and hypoketonemia. Our observations also suggest a critical role of PPARa in the up-regulation of these metabolisms in chicken liver in response to fasting. First, we observed a marked induction of *PPARa *expression after 16 h and 48 h of fasting, confirming earlier results by Cogburn et al [23] in the same species. Second, we identified potential PPRE in avian genes *ACOX1, CPT1 *and *HMGCS2 *involved respectively in peroxisomal and mitochondrial beta-oxidation and ketogenesis. Third we observed a significant correlation between *PPARa *and *ACOX1, EHHADH, CPT1 *mRNA levels. Fourth, within the 15 genes showing the highest level of induction following 16 h of fasting (see additional file 1: cluster 2), we found 8 genes involved in fatty acid beta-oxidation (*PECI, ACAA1, ACOX1, CPT1A, HADHA*), ketogenesis (*HMGCL, ACAT1*), or gluconeogenesis (*PCK1*), the majority being direct targets of PPARa.

By contrast, hepatic expression of *SREBP1 *(encoding a key transcription factor controlling the expression of lipogenic enzymes in mammals) decreased after the first 16 h of fasting. We also observed a significant correlation between *SREBP1 *mRNA levels and those of their putative target genes *ACLY, ACACA, FASN *and *SCD*. These results suggest a key role of SREBP1 in the regulation of fatty acid synthesis in chickens as already suggested by others studies carried out on the same species [40,41] and demonstrated in rodents [37,38,42] using 24 h of fasting.

SREBP2 regulates the expression of many genes involved in cholesterol synthesis. Cholesterol is an essential component of animal cell membranes, and its concentration is tightly controlled by a feedback system that operates at transcriptional and posttranscriptional levels [43]. In the present study, we observed a decrease of *SREBP-2, HMGCR, LSS *and *CYP51A1 *expressions during food deprivation. HMGCR is considered as the rate limiting enzyme of cholesterol synthesis. These results suggest a decrease of cholesterol synthesis in response to starvation in chickens, as already reported in rodents after 24 h or 48 h of fasting [10,14,44]. However, HMGCS1 gene another gene involved in cholesterogenesis exhibited a strikingly different pattern of expression, with a marked induction at 16 h of fasting which is contrast with the repression of *HMGCS1 *expression observed during starvation in mice and pigs [10,12]. Two distinct genes condense acetyl-CoA with acetoacetyl-CoA to form HMG-CoA in the cell, the first one HMGCS2, located in mitochondria is involved in ketogenesis, whereas the second one, HMGCS1 controls cholesterogenesis in the cytosol. Like in other species, these two genes have been characterized in avian species [45], allowing us to design gene specific oligonucleotides. Four isoforms of the HMGCS1 enzyme have been described in chicken liver [46] while only one was characterized in rat liver. The specific role of the 4 isozymes and the mechanism to generate them are not clear and must be clarified to better understand the species difference in *HMGCS1 *expression.

NR1H3 (named also LXRa) is a transcription factor that belongs to the nuclear hormone receptor family and was first discovered as playing a critical role in cholesterol homeostasis and bile acid metabolism through the regulation of Cyp7a1 (cholesterol 7 hydroxylase), the rate-limiting enzyme of cholesterol degradation to bile acids [47] It is also a master lipogenic transcription factor, directly regulating *SREBP1 *[48,49], *FASN *[50], *SCD *[51] and *ChREBP *genes [52]. We therefore expected its expression profile to parallel to those of its potential avian targets during the feeding-fasting transition. Curiously, we observed that *NR1H3 *mRNA level was increased at 16 h of fasting and returned to fed levels by 48 h. This result is consistent with a previous study conducted in rodents: rats fasted for 24 h increased LXRa mRNA level by 3-fold [53]. The role of NR1H3 during starvation remains to be elucidated.

We then studied the effects of fasting on the hepatic expressions of the three genes encoding the desaturases SCD, FADS1 and FADS2. SCD (also named delta9-desaturase) catalyzes the synthesis of monounsaturated fatty acids (MUFAs) whereas FADS1 (delta5-desaturase) and FADS2 (delta6-desaturase) are two key enzymes for the synthesis of highly polyunsaturated fatty acids (HUFA). All three mammalian desaturases are induced at the mRNA levels by PPARa [54-56] and SREBP1 [57-59]. This dual activation is a specificity of the desaturases compared to the enzymes of beta-oxydation or lipogenesis which are exclusively regulated by either PPARa or SREBP1 (for review, see [60]). In rodents, *SCD *gene activity is completely repressed during fasting, like the activity of other lipogenic genes (*ACLY, ACACA, FASN*) and *SREBP1 *[37,38,61]. We observed the same regulation for the chicken *SCD *gene: the first 16 h of fasting resulted in a 14-fold inhibition of its expression and lasted up to 48 h of fasting. Because of their common regulation by SREBP1 and PPARa, similar modulations could be expected for the three genes in response to fasting. By contrast, we observed that *FADS1 *and *FADS2 *genes were only significantly repressed after 48 h of fasting (4- or 6-fold). In pig liver, *SCD *and *FADS1 *were down-regulated after 24 h of fasting [12]. To our knowledge, no data about the *FADS1 *and *FADS2 *expression modulation in response to fasting are available in rodents. This difference of modulation between *SCD *gene and the two genes *FADS1 *and *FADS2 *in response to short-fasting suggest an additional regulatory mechanism between SCD1 and these two latter genes. Such a hypothesis was already done by Matsuzaka et al [58], from their data obtained during fasting-refeeding treatment in which no changes of *FADS1 *and *FADS2 *expression contrary to the lipogenic genes was observed. So, these two enzymes which play crucial roles in the production of HUFA, might have an important role in fasted state because HUFA were reported to inhibit SREBP-1 activity by multiple mechanism [62-65] and to be ligands of PPARa.

## Conclusion

In the present study, we successfully used a chicken 20 K oligo microarrays to analyze the alteration of hepatic gene expression profile upon starvation. We identified 1162 genes differentially expressed between the 16 h or 48 h fasting states and the fed state. We provide a valuable and publicly available resource of genes profiles altered during the first 48 h of starvation in chicken liver. After 16 h of fasting we observed an up-regulation of genes involved in fatty acid oxidation, ketogenesis, gluconeogenesis and a down-regulation of genes involved in fatty acid and cholesterol synthesis, which is consistent with earlier results obtained in mammals. After 48 h of fasting, when the number of genes showing an altered expression was much higher (about 3.5-fold higher), the annotation data suggest a repression of genes involved in numerous signalling pathways. As a whole, we observed that more genes were down-regulated than up-regulated in response to starvation. The expression profiles of candidate genes encoding key transcription factors and enzymes involved in lipid metabolism but not present on the microarray were evaluated by qRT-PCR. The results were similar to those reported in mammals except for the gene HMGCS1, which was induced at 16 h of fasting in chicken liver and repressed in mouse and pig liver. Our data also suggest that the genes *SCD*, *FADS1 *and *FADS2 *encoding different desaturases are regulated differently during fasting and *NR1H3 *is up-regulated at 16 h of fasting while one of its target, *SREBP1 *is down-regulated. Further studies should be performed to precise their role in the complex regulation array of lipid metabolism.

## Methods

### Animals and experimental procedures

Male broiler chicks obtained from a commercial hatchery were bred at INRA, UR083, Recherches Avicoles, F-37380 Nouzilly in accordance with European Union guidelines for animal care and under the authorization 006621 delivered to M.J. Duclos by the French Ministry of Agriculture. All birds were reared up to 1 week of age in floor pens, then raised for 2 weeks in cages under "14 h light:10 h darkness" cycles. During this period, they were fed a balanced starter diet (12.12 MJ metabolized energy/kg containing 22% crude protein). At 3 weeks of age, the chickens were weighed and assigned to seven experimental groups (n = 10 per group), equalizing body weight and variance between groups. One week later, each group was submitted to one of the following treatments: fed ad libitum (Fed), fasted for 16 h or 48 h (Fst16 and Fst48, respectively), and four other treatments not analyzed in this present study [66]. All birds were given free access to water at all time. Following sacrifice, liver were collected, quickly frozen into liquid nitrogen and stored at -80°C until molecular analysis.

### RNA isolation

Total RNA was extracted with TRIzol^® ^reagent (Invitrogen, Cergy Pontoise, France) according to the manufacturer's instructions. Quality and concentration of extracted RNA were assessed using a 2100 Bioanalyzer (Agilent Technologies, Massy, France).

### Array slides and annotation

The chicken 20 K array was obtained from ARK-Genomics (Roslin institute-UK: ). The array design has been published in the ArrayExpress repository with the accession A-MEXP-820 [67] and in Gene Expression Omnibus with the platform name GPL5480 [68].

Briefly, the DNA microarray was produced from 20,460 oligonucleotides (whose the size varies from 60 to 75 nucleotides) designed using the OligoArray 2.0 software against the chicken ENSEMBL transcripts. The transcripts were selected from the chicken genome draft available in december 2004 and extensive matching of the UMIST and DT40 full length EST's with the TIGR clusters. Because the 20 K oligonucleotide set was defined in 2004–2005 from heterogeneous data sources, we checked the quality of the previously designed oligos, comparing them with the chromosomes of the 2.1 Washington University assembly of the chicken sequence genome [69]. The comparison was made using NCBI Blast with a 75% similarity threshold over 50 base pairs. Then for each high scoring pair (HSP) we retrieved the transcripts corresponding to the location using the Ensembl API (version Ensembl 43). An oligonucleotide had to be in a unique gene (even if it was spanning 2 exons) to be selected for further analyses. The corresponding annotations were then retrieved from Ensembl using the blast HSP coordinates. As results, among the 20460 gene-oligonucleotides, 13057 were identified as aligning with a unique coding region in the chicken genome sequence. As we retrieved an Ensembl gene name and/or a GO biological process term for only 32% of the 13057 oligo sub-set, we decided to rely on human orthologs (according to the "one to one" criteria of ENSEMBL annotation) which could be identified for 81% of the 13057 oligonucleotides, allowing to retrieve HGNC-HUGO gene symbol for the majority of them (70% of 81% of 13057). The annotations obtained by a bioinformatics procedure developed by SIGENAE (INRA) are available on the web site: [69]. Finally, of the 13057 oligonucleotides, 7419 presented a validated HUGO gene symbol from which we could extract more GO terms from GOA human annotation database using the two softwares "Hugo my Genes" and "GOret" developed by the Rennes transcriptome platform: .

### Microarray procedures

#### mRNA labelling and hybridization

Five μg of each RNA sample were reverse-transcribed and Cy5 fluorescent-labelled using the ChipShot™ Direct Labeling kit (Promega, Charbonnieres, France). Each Cy5-labelled RNA was hybridized to the microarray with a same Cy3-labelled reference probe according to the Transcriptome-Biochips Platform of Genopole "Toulouse Midi-Pyrénées" (France) procedure. Briefly, experiments were carried out with an automatic hybridization chamber (Discovery from Ventana Medical System, Inc). Prehybridization was carried out with a freshly prepared solution of 1% BSA, 2× SSC, 0.1% SDS over 30 min at 42°C. After automatic washing according to manufacturer's instructions, the slides were hybridized for 8 h in 200 μl of ChipHybe™ buffer (Ventana Medical System, Inc) containing 10 μl of labelled cDNA purified. After hybridization, the slides were washed twice for 2 min in 2× SSC/0.1% (v/v) SDS, and for 2 min in 0.1× SSC. We finally obtained 9, 7 and 7 microarrays respectively for the three nutritional conditions Fed, Fst16 and Fst48. For each gene, the fluorescence ratio reflected the relative abundance of the mRNA of interest in each experimental sample compared with the same reference mRNA. The reference allowed thus to take into account an eventual "spot × array" interaction.

#### Data acquisition

Detection of the fluorescence signals was made with a laser scanner (GenePix 4000A from Axon Instrument, CA) keeping a constant PMT gain for each channel. The images were then analyzed with GenepixPro 4.0 software (Axon instruments, Inc., Union City, CA). Row data file for each array containing all measured values were stored in genepix files compatible with the LIMMA library of R-project statistical and Bioconductor environment [70] which was used for the normalization and the analysis of the data.

#### Filtering for data normalization

For the normalization step, data were filtered according to 3 criterions: i) the genepix flag criterion automatically performed by GenepixPro 4.0 [71], ii) the SNR (Signal to Noise Ratio) provided also by GenepixPro and which was set to 2, iii) a asymmetry criterion of the spot which was set to 20%. For all microarrays, the mean percentages of spots discarded for the genepix flag, SNR and asymmetry criterions were 3.3%, 10.4% and 3.7% respectively. It was 16% for the three criterion taken together, showing the good quality of all technical procedures from slide production to labelling and hybridization. However, 5 arrays (3 and 2 in Fed and Fst16 conditions respectively) which presented a percentage of spots not conformed to the SNR criterion higher than 50% were excluded from subsequent analysis. These 5 microarrays were discarded among a set of 60 microarrays corresponding to a larger experimental design with 6 treatments, confirming a global good quality of the technical procedures. The homogeneity of the background was systematically checked on each microarray by the boxplot and imageplot procedures of the LIMMA package. We finally analyzed 18 microarrays: 6, 5 and 7 microarrays for Fed, Fst16 and Fst48 conditions respectively.

#### Data normalization

The ratio Cy5/Cy3 used was expressed as the Log2 of the ratio of median pixel intensity of the two red and green spots. Log2 median ratio values were then normalized on each individual array (ratio centered on zero) according to the hypothesis that the majority of gene expressions do not differ between two samples. The centering was performed by "Lowess fitness" [72] to take into account the intensity dependence of the fluorescence bias. Only the array spots conform to the filtering step were used for the normalization.

#### Data analysis

All data analyses were performed using the R project statistical and Bioconductor environment [70]. Analysis of variance using the "eBayes" procedure [24] and analysis of contrasts were performed with LIMMA library appropriated to bi-color genepix files. Two-way Hierarchical Cluster Analysis (HCA) was performed using hclust function with "1-cor" as distance and "ward" as aggregation criterion; "heatmap" function was used to generate images. Principal Component Analysis (PCA) was performed with FactoMiner library. In this study, we were interested in comparing the two Fst16 and Fst48 conditions with the Fed state. The significance of gene expression differences between the nutritional conditions was assayed by analysis of variance followed by an analysis of the two contrasts "Fst16-Fed" and "Fst48-Fed". Because of the high test number (13057) needing an appropriated control of the false positive rate, the pvalue of each gene for each contrast was corrected according to the false discovery rate (FDR) procedure of Benjamini-Hochberg [25]; the FDR is the expected proportion of erroneously rejected null hypotheses among the rejected ones. Gene expression difference was declared significant if its corrected pvalue was p < 0.01. Results were further filtered by retaining only the genes exhibiting at least an amplitude of variation higher than +/- 40% between two nutritional states of interest ((absolute(log2(ratio)) > 0.485).

### Gene ontology, KEGG and IPA analyses

Gene ontology (GO) constitutes a controlled vocabulary of about 20,000 terms organized in three independent hierarchies for cellular components, molecular functions, and biological processes [73]. Gene ontology analyses of clusters identified by HCA were performed using the Gene Ontology Tree Machine (GOTM) software [74]. Hypergeometric test was used as the statistical method to select enriched biological process GO terms for each cluster compared to the GO terms of the annoted genes present on the microarray (7419 genes). The biological process GO terms were considered as enriched for a level of pvalue < 0.01. The biological interpretation of the gene clusters were further completed by KEGG annotation [26] and Ingenuity Pathway Analysis, the latter software using various annotation data (IPA, Ingenuity Systems Inc., Redwood City, CA). Were only conserved Kegg pathways with a minimum of 3 genes associated and having a probability to be observed in the cluster 4-fold superior than the probability to obtain it by chance. For Ingenuity pathways, we only reported in the present study the top five canonical pathways having a pvalue < 0.01 and at least 3 genes affiliated.

### Real time quantitative RT-PCR (qRT-PCR) assay

A set of 11 genes present on the microarray was chosen for confirmation by quantitative RT-PCR, 8 of which were significantly differentially expressed between the 16 h fasting state and the Fed state. In addition, 8 candidate genes not present on the microarray were also measured by qRT-PCR (Figure 4).

Reverse transcription (RT) was carried out using the high-capacity cDNA archive kit (Applied Biosystems, Foster City, CA) according to the manufacturer's protocol. Briefly, 200 μL of each reaction mixture containing 20 μL of 10× RT buffer, 8 μL of 25X dNTPs, 20 μL of 10X random primers, 10 μL of MultiScribe Reverse Transcriptase (50 U/μL), and total RNA (10 μg) was incubated for 10 min at 25°C followed by 2 h at 37°C. A 1/10 or 1/20 dilution, dependant on the gene, of each RT reaction was further used for real time quantitative PCR (qPCR). cDNA samples were mixed with 20 μl ABsolute SYBR Green Mix (Abgene, UK) and 300, 450 or 600 nM, according to the gene, of specific reverse and forward primers (Table 3). Reaction mixtures were incubated in an iCycler iQ Multicolour Real-Time PCR Detector (Bio-Rad, Marne la Coquette, France) programmed to conduct one cycle (95°C for 15 min) and 40 cycles (95°C for 15 s and 59°C for 45 s). A melting curve program was then performed for each gene to check the presence of an unique product with specific melting temperature. For each sample and each gene, PCR runs were performed in triplicates. For each gene, serial PCR reactions constructed with 2-fold serial dilutions from a pool of the cDNA samples were systematically added on each microplate for the calibration curve and determination of the amplification rate (R) of the Taq polymerase. For all genes including *18S*, the amplification rates were in a range of 99% to 100% and could be considered as equal to 1. So, for a same sample, the gene expression level could be normalized relative to the 18S expression level as follows: Gene normalized CT = CTgene – CT18S = ΔCt. The significance of expression differences between nutritional states were analyzed by analysis of variance and analysis of contrasts on the basis of the gene normalized CT values using AOV package of R environment. For each gene, the N-fold gene expression difference between two conditions (1 versus 2) was expressed as: Fold change = 2 exp(-ΔΔCt) with ΔΔCt = mean(ΔCt) 2- mean(ΔCt) 1, where (ΔCt)i are the mean of the gene normalized Ct of the different samples of the condition i.

**Table 3:** Selected qRT-PCR primer sequences and accession numbers

**Gene symbol**	**Gene name**	**Ensembl or Gene bank Accession number**	**Primer sequence (forward/reverse)**
ACACA	Acetyl-Coenzyme A carboxylase alpha	ENSGALG00000005439	GAGGAGGGAAGGGAATTAGGAA CCAAGTGGCGGGACTGTT

**ACOX1**	Acyl-Coenzyme A oxidase 1	ENSGALG00000002159	TCATCCGGTCTCTGATTGTAGGA GCACTATAGCGGATGGCAATG

**ACLY**	ATP citrate lyase	ENSGALG00000003475	GGCGTGAATGAACTGGCTAAC TAGTCTTGGCATAGTCATAGGTCTGTTG

**CPT1A**	Carnitine palmitoyltransferase1A	ENSGALG00000007077	CCCTGAAAATGCTGCTTTCCTA TGGTGCCTGCAGAAAGTTTG

**CPT2**	Carnitine palmitoyltransferase II	ENSGALG00000010681	CCTGAACGCCCAGAAACCT CCCTTTTCAAACTGATGAGCAAGT

CYP7A1	Cytochrome P450, family 7, subfamily A, polypeptide 1	ENSGALG00000015432	TGATGACATGGAAAAAGCAAAGA CCAAAAAGTAGCAGGAATGGTGTT

**EHHADH**	Peroxisomal bifunctional enzyme	ENSGALG00000006680	TCATAGAAAGGAGCGAGAAGC AGCAGGAACCCCAACCAGT

FADS1	Fatty acid desaturase 1	ENSGALG00000007127	CAGCACCACGCGAAACC TCTACAGAGAGCTTCTTTCCCAAAG

FADS2	Fatty acid desaturase 2	ENSGALG00000007178	CCATGATCAAGCGCAGGTT ATGTATGTGATGAAATAGCGCATGTAG

**FASN**	Fatty acid synthase	ENSGALG00000002747	TGAAGGACCTTATCGCATTGC GCATGGGAAGCATTTTGTTGT

**HMGCS1**	Hydroxymethylglutaryl-CoA synthase, cytoplasmic	ENSGALG00000014862	GCTGGTGCTGTTGCTATGCT TGTCTGTCCCCTCTTTTTGC

**HMGCS2**	Hydroxymethylglutaryl-CoA synthase, mitochondrial	ENSGALG00000002960	GGTGGTGTGTGGGGACAT GGTAGCACTGGATGGAGAGG

**HMGCR**	3-hydroxy-3-methylglutaryl- Coenzyme A reductase	ENSGALG00000014948	CTGGGTTTGGTTCTTGTTCA ATTCGGTCTCTGCTTGTTCA

NR1H3	Nuclear receptor subfamily 1, group H, member 3 (LXRα)	ENSGALG00000008202	TCCCACTCAACTCAGCACAC CAGACTTCATTTCCCAGCATC

**PCK1**	Phosphoenolpyruvate carboxykinase, cytosolic	ENSGALG00000007636	CTGCTGGTGTGCCTCTTGTA TTCCCTTGGCTGTCTTTCC

PPARA	Peroxisome proliferative activated receptor, alpha	ENSGALG00000022985	AGCATCCAGTCCTTCATCCA AAAAACCCTTACAACCTTCACAA

**SCD**	Stearoyl-CoA desaturase(delta-9-desaturase)	ENSGALG00000005739	TTTGGCAATCGGCCGTAT TGGTAGTTGTGGAAACCTTCTCCTA

SREBF1	Sterol regulatory element binding protein 1	gb:AY029224	GTCGGCGATCCTGAGGAA CTCTTCTGCACGGCCATCTT

SREBF2	Sterol regulatory element binding protein 2	ENSGALG00000011916	GGCTGGCTTCTCCCCCTAT GTTCATCCTTAACCTTTGCATCAT

Genes in bold were present on the microarray.

### PPRE prediction in the HMGCS2, CPT1A and ACOX1 genomic DNA sequences

Several custom PERL scripts were developped to automate the PPRE detection procedure. Gallus gallus (GGA) genomic DNA sequences (including 5000 bp upstream and 3000 bp downstream sequence from the start of the first exon) were extracted from the Ensembl website by the GGA Ensembl ID. The orthologous genomic sequences of Homo sapiens and Mus musculus were automatically extracted (1-to-1 ortholog type) using the Compara API [75]. The DNA sequences were analyzed by the STAN [76] and the PATSER software [77]. The PPRE patterns used for every gene were designed from previous results found in the literature [78-82]. For STAN PPRE detection, one degree of freedom were applied on each part of the DR1 (e.g., for HMGCS2, the PPRE pattern syntax was: "AGACCT":1, 1...1, "TGGCCC":1, see Nicolas et al. [76] for details on the syntax used in STAN). For PATSER detection, Positional Weight Matrix (PWM) were generated using CONSENSUS software [77] using PPRE sequences described by previous studies. Raw results obtained by both softwares were post-analyzed by another custom PERL script.

## Abbreviations

PPARa: Peroxysome Proliferators-Activated Receptor alpha; FADS1: Fatty Acid Desaturase 1 (delta5-desaturase); FADS2: Fatty Acid Desaturase 2 (delta6-desaturase); SCD: Stearoyl CoA Desaturase 1 (delta9-desaturase); SREBP1 and 2: Sterol Regulatory Element Binding Protein 1 and 2; NR1H3: Nuclear Receptor Subfamily 1, group H, member 3 (noted LXRa for Liver X receptor alpha); HMGCS1: 3-Hydroxy-3-MethylGlutaryl-CoA Synthase 1; ME: Malic Enzyme; ACLY: ATP Citrate Lyase; ACACA: Acetyl CoA Carboxylase; CPT1A: Carnitine PalmitoylTransferase 1A; EHHADH: Enoyl-CoA Hydratase/3-HydroxyAcyl CoA DeHydrogenase; PEPCK: PhosphoEnolPyruvate CarboxyKinase; PPRE: PPAR Element; GO: Gene Ontology; KEGG: Kyoto Encyclopedia of Genes and Genomes; qRT-PCR: quantitative Reverse Transcription-Polymerase Chain Reaction; HGNC: HUGO Gene Nomenclature Committee; FDR: False Discovery Rate.

## Authors' contributions

CD and SL were responsible for generating the gene expression data and for interpretation and analysis of the results. CD and CB carried out the qRT-PCR experiments. PB, FH and SL performed the statistical analyses with R. FL performed the PPRE prediction. MJD designed and provided the animal experimental design, contributed advice on the analysis and critically reviewed the manuscript. FM, CK, MA and SL contributed to the annotations of the 20 K microarray. PLR and SL defined the microarray experimental design. CD and SL drafted the manuscript. MD, CDi, and CB reviewed the manuscript. SL proposed and supervised the research. All authors read and approved the final manuscript.

## Supplementary Material

Additional file 1 **List of the 1162 genes differentially expressed in the chicken liver after 16 h or 48 h of starvation ordered according to their HCA cluster**. These 1162 genes i) were significantly differentially expressed between fasting and fed states (pvalue < 0.01), ii) exhibited at least an expression difference between two conditions exceeding an absolute 1.4 fold ratio, iii) were annoted through their human ortholog. The selected 0.01 pvalue that was corrected according to the false discovery rate (FDR) of Benjamini-Hochberg [25] ensures in average a number of false positive of 12 genes in the selected gene set. For each cluster, genes were ordered by decreasing log2(ratio) of the "Fst16-Fed" contrast for the clusters 1 and 2 and then of the "Fst48-Fed" contrast for the clusters 3 and 4. Click here for file
